# Development and validation of an index to assess hospital quality management systems

**DOI:** 10.1093/intqhc/mzu021

**Published:** 2014-03-11

**Authors:** C. Wagner, O. Groene, C. A. Thompson, N. S. Klazinga, M. Dersarkissian, O. A. Arah, R. Suñol, on behalf of the DUQuE Project Consortium, N Klazinga, DS Kringos, MJMH Lombarts, T Plochg, MA Lopez, M Secanell, R Sunol, P Vallejo, P Bartels, S Kristensen, P Michel, F Saillour-Glenisson, F Vlcek, M Car, S Jones, E Klaus, S Bottaro, P Garel, M Saluvan, C Bruneau, A Depaigne-Loth, C Shaw, A Hammer, O Ommen, H Pfaff, O Groene, D Botje, C Wagner, H Kutaj-Wasikowska, B Kutryba, A Escoval, A Lívio, M Eiras, M Franca, I Leite, F Almeman, H Kus, K Ozturk, R Mannion, OA Arah, A Chow, M DerSarkissian, CA Thompson, A Wang, A Thompson

**Affiliations:** 1 NIVEL, Netherlands Institute for Health Services Research, Utrecht, the Netherlands; 2 Department of Public and Occupational Health, EMGO Institute for Health and Care Research, VU University Medical Center, Amsterdam, theNetherlands; 3 Department of Health Services Research and Policy, London School of Hygiene & Tropical Medicine, London, UK; 4 Department of Epidemiology, Fielding School of Public Health, University of California, Los Angeles (UCLA), Los Angeles, CA, USA; 5 Palo Alto Medical Foundation Research Institute, Palo Alto, CA, USA; 6 Department of Public Health, Academic Medical Center, University of Amsterdam, Amsterdam, theNetherlands; 7 UCLA Center for Health Policy Research, Los Angeles, CA, USA; 8 Avedis Donabedian Research Institute (FAD), Universitat Autonoma de Barcelona, Catalonia, Spain; 9 Red de investigación en servicios de salud en enfermedades crónicas (REDISSEC), Barcelona, Spain

**Keywords:** quality management, hospital care, surveys, patient safety, health care system

## Abstract

**Objective:**

The aim of this study was to develop and validate an index to assess the implementation of quality management systems (QMSs) in European countries.

**Design:**

Questionnaire development was facilitated through expert opinion, literature review and earlier empirical research. A cross-sectional online survey utilizing the questionnaire was undertaken between May 2011 and February 2012. We used psychometric methods to explore the factor structure, reliability and validity of the instrument.

**Setting and participants:**

As part of the Deepening our Understanding of Quality improvement in Europe (DUQuE) project, we invited a random sample of 188 hospitals in 7 countries. The quality managers of these hospitals were the main respondents.

**Main Outcome Measure:**

The extent of implementation of QMSs.

**Results:**

Factor analysis yielded nine scales, which were combined to build the Quality Management Systems Index. Cronbach's reliability coefficients were satisfactory (ranging from 0.72 to 0.82) for eight scales and low for one scale (0.48). Corrected item-total correlations provided adequate evidence of factor homogeneity. Inter-scale correlations showed that every factor was related, but also distinct, and added to the index. Construct validity testing showed that the index was related to recent measures of quality. Participating hospitals attained a mean value of 19.7 (standard deviation of 4.7) on the index that theoretically ranged from 0 to 27.

**Conclusion:**

Assessing QMSs across Europe has the potential to help policy-makers and other stakeholders to compare hospitals and focus on the most important areas for improvement.

## Introduction

In a recent review on instruments assessing the implementation of quality management systems (QMSs) in hospitals, the authors conclude that hospital managers and purchasers would benefit from a measure to assess the implementation of QMS in Europe. The results of the review show that there is currently no well-established measure that has also be used to assess the link between quality management at hospital level, quality management activities at departmental level and patient outcomes [1]. In the context of the European cross-border directive and the Council Recommendation on patient safety, it is even more important to measure and compare the implementation of QMS across countries to get insight into existing prerequisites for safe patients' care and possible gaps in the quality management within or between countries. QMSs definition used in this article is ‘as a set of interacting activities, methods and procedures used to monitor, control and improve the quality of care’ [2].

In the recent review, 18 studies to assess the implementation of QMSs have been described. Only nine of these studies reported methodological criteria in sufficient detail and were rated as good [1]. Only two of them have been used in several European countries, e.g. the European Research Network on Quality Management in Health Care (ENQuaL) questionnaire for the evaluation of quality management in hospitals [3, 4], and the Methods of Assessing Response to Quality Improvement Strategies (MARQuIS) questionnaire and classification model for quality improvement systems [5]. Despite their good evaluation, both instruments have important limitations. The ENQuaL questionnaire was developed in 1995 and does not cover more recent quality management topics, such as ‘use of indicator data’ and ‘learning from adverse events’ [2]. The MARQuIS questionnaire is very long (113 items) and focusses mainly on leadership (36 items), policy, planning, documents (20 items), quality strategies (laboratory) (20 items) and structure (19 items), but less on the evaluation of care processes by indicator data. The latter is an important step in the quality improvement cycle [5].

The objective of this study was to develop and validate an up-to-date and more concise survey instrument to assess hospital QMSs in European countries, and to compute an index—the Quality Management Systems Index (QMSI)—representing its developmental stage. Specifically, we report on its structure, reliability, validity and descriptive statistics of the QMSI and its scales.

## Methods

### Conceptual considerations

A broad range of activities can be used by an organization to maintain and improve the quality of care they deliver. These activities might change over time because of new evidence, changing expectations of the public or new (national) regulations regarding accountability. When developing a multi-item measurement instrument, we need to know the underlying relationship between the items (quality activities) and the construct to be measured, e.g. the QMS. The new instrument has, like the earlier developed ENQuaL and MARQuIS questionnaire, partly been based on the nine enabler and resulting themes of the existing theoretical framework of the European Foundation of Quality Management model (EFQM) [6], but some of the questionnaire items had to be changed to represent actual developments in quality management practice.

### Development of the instrument

**Figure 1:**
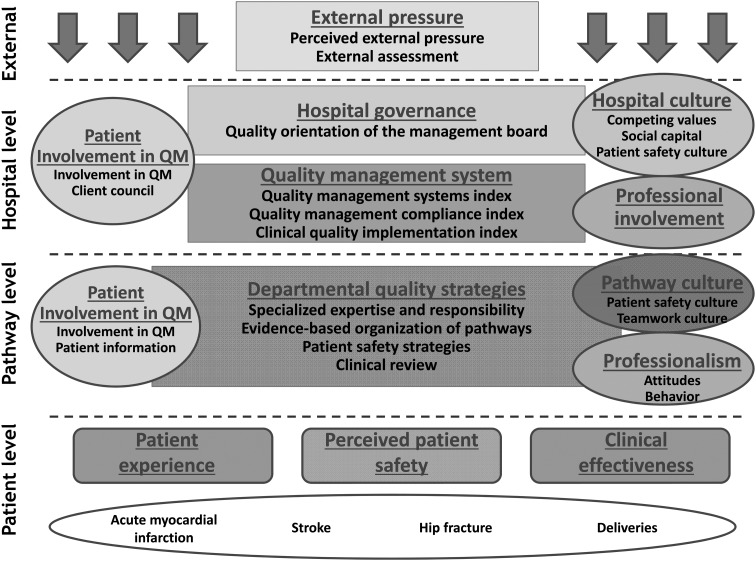
Conceptual model of DUQuE.

The questionnaire was a web-based multi-item and multi-dimensional instrument to assess the development of QMSs in hospitals. The aim of the questionnaire was to focus on the managerial aspects of quality management such as policy documents, formal protocols, analyzing performance and evaluating results, and not on leadership, professional and patient involvement or organizational culture, as these are different theoretical concepts within the Deepening our Understanding of Quality improvement in Europe (DUQuE) framework (Fig. 1) and which are assumed to influence the implementation of QMS. The literature review revealed that earlier studies have distinguished six domains of quality management, e.g. procedures and process management, human resource management, leadership commitment, analysis and monitoring, structures and responsibilities and patient involvement. Most of the instruments do not cover all the domains. If they do, they have a large number (179) of items [1].

Several steps were applied to develop a more concise instrument that still covers the most important domains of QMS presented in the literature.

To select items from earlier questionnaires (ENQuaL and MARQuIS) [2, 5] or develop new items for the DUQuE questionnaire, we first used the expert opinion of other DUQuE project members. They considered the most relevant and possibly most influencing activities for the improvement of patient-related outcomes (*n* = 10). The experts have a long history in healthcare, especially in quality management in the various countries. For a concise instrument, only the most relevant activities are important. Second, items related to the more managerial focal areas of the theoretical framework of the EFQM model were selected (policy documents, human resources, processes and feedback of results such as patient and professional experience, comparison of clinical and societal performance). The wording of the items and the answer categories were compared with accreditation manuals and the review on existing instruments. In the end, most questionnaire items for the new instrument came from the ENQuaL and MARQuIS questionnaire, but because of our focus on managerial aspects of quality management, not all focal areas of these instruments were selected. Finally, the answer categories of all items were standardized with a focus on the extent of implementation and four answer categories.

The questionnaire was first developed in English and was translated into seven languages using a forward–backward translation process for validation. Respondents could rate each item on a four-point-Likert-type scale, with answer categories ranging from ‘Not available’ to ‘Fully implemented’ and from ‘Disagree’ to ‘Agree’.

The content validity of the final questionnaire used in the DUQuE project was approved and judged completely by the 10 experts from different quality research areas involved in the project who were not involved in the Quality Management Systems Index **(**QMSI) development. The questionnaire consisted of 56 items divided over 5 dimensions: quality policy (10 items), quality resources (9 items), performance management (7 items), evidence-based medicine (13 items) and internal quality methods (17 items).

### Setting and participants

The study took place within the context of the DUQuE project that ran from 2009 to 2013 [7, 8] in 7 European countries: France, Poland, Turkey, Portugal, Spain, Germany and Czech Republic. These countries represent the diversity of Europe (e.g. countries from the East/West, North/South, regional/national healthcare system, system in transition/longer established system). In each country, 30 hospitals were randomly recruited if they had >130 beds and were treating patients with acute myocardial infarction, hip fracture and stroke and handled child deliveries. The conditions were chosen for their high financial volume, high prevalence of the condition and percentage of measureable complications, and the different types of patients and specialists they were covering [7, 8]. Of the 210 approached hospitals, 188 were able to participate (89.5% response rate). The DUQuE QMSI questionnaire was administered online to the quality managers of the 188 participating hospitals (response *N* = 183; 97%). A quality manager of a hospital was defined as the person who is responsible for the coordination of quality improvement activities. He/she should have a good overview of all activities toward quality improvement (questionnaire instruction). The quality manager was allowed to ask other people in the hospital if he/she was not sure about the right answer, but only one questionnaire per hospital was expected to be filled in. The instruction also said that it was not necessary for a hospital to have all activities mentioned in the questionnaire and that it was expected that hospitals would be in different phases of implementation for different activities.

Ethical approval was obtained by the project coordinator at the Bioethics Committee of the Health Department of the Government of Catalonia (Spain).

#### Data collection

Respondents who participated in the DUQuE project were invited by a letter and personally by the country coordinator. Questionnaires were completed anonymously and directly entered in the online data platform. The data were collected between May 2011 and February 2012. All participants were sent passwords to access the web-based questionnaire and sent reminders.

#### Statistical analyses

We began by describing the hospitals and quality managers that provided responses to the main questionnaire used to develop the index. Next, we used psychometric methods to investigate the structure, reliability and validity of the QMSI instrument. We assumed that our ordinal data approximated interval data and conducted exploratory factor analysis and confirmatory factor analysis, reliability coefficient, item-total scale correlation and inter-scale correlation analyses [9–11]. These were done separately for each of the theoretical themes. We explored the factor structure of the questionnaire using split-file principal component analysis with oblique rotation and an extraction criterion of eigenvalues of >1 while requiring three or more item loadings. Items were grouped under the factor or scale where they displayed the highest factor loading. Only items that had loadings of at least 0.3 were assigned to a factor [10]. Confirmatory factor analysis was then used on the second half of the sample to determine whether the data supported the final factor structure. A root mean square residual of <0.05 and a non-normed fit index of >0.9 indicated good fit of the scale structure to the data. We then performed reliability analysis using Cronbach's alpha where a value of 0.70 or greater indicated acceptable internal consistency reliability of each scale [12, 13]. We also examined the homogeneity of each scale using item-total correlations corrected for item overlap. Item-total correlation coefficients of 0.4 or greater provided adequate evidence of scale homogeneity. Finally, we assessed the degree of redundancy between scales by estimating inter-scale correlations using Pearson's correlation coefficients, where a correlation coefficient of <0.7 indicated non-redundancy [11, 14].

Once we had a final factor structure, we computed the score for each of the scales by taking the mean of items used to build the scale. We used appropriate multiple-imputation techniques to handle missing data for hospitals with missing data for four or fewer scales used to build the final QMSI [15]. The scores of the extracted scales of our analysis were then summed in order to construct the final QMSI. We subtracted the number of factors or scales from this sum in order to bring the lower bound of the scale down to zero.

In order to validate our instrument, we further examined correlations with two other measures of quality management based on on-site visits by external auditors. These other constructs were the Quality Management Compliance Index (QMCI) and the Clinical Quality Improvement Index (CQII) [16]. The QMCI measures the compliance of healthcare professionals, managers or others responsible in the hospital with quality management strategies. The CQII measures the implementation of clinical quality strategies by healthcare professionals. Both measures are based on on-site visits of external auditors and are described separately in this supplement [16].

We used Pearson's correlation coefficients to assess the relationship between QMSI, QMCI and CQII, deeming coefficients between 0.20 and 0.80 as acceptable [10, 14, 17]. If the QMSI measures the implementation of QMS in hospitals, it is expected that there would be a positive non-collinear relationship between the QMSI and the two more independent measures of quality management: QMCI and CQII. Because only some parts of the content of the three instruments overlap, the coefficients will not be very high.

All statistical analyses were carried out in SAS (version 9.3, SAS Institute, Inc., NC, USA. 2012).

## Results

### Participants

A total of 188 hospitals participated in the DUQuE project. Quality managers of all the hospitals responded to the questionnaire, but five quality managers provided not enough data to calculate the nine scales and the QMSI. Background characteristics of the participating hospitals and the quality managers who filled in the questionnaire are given in Table 1.

**Table 1:** Characteristics of hospitals and quality managers (*N* = 183)

Hospital characteristics	
Teaching status, *N* (%)	
* *Non-teaching	106 (57.9)
Teaching	77 (42.0)
Ownership, *N* (%)	
Private	32 (17.4)
Public	151 (82.5)
Number of beds, *N* (%)	
<200	18 (9.8)
200–500	78 (42.6)
500–1000	60 (32.7)
>1000	27 (14.7)
Quality manager characteristics	
Sex, *N* (%)	
Male	60 (32.7)
Female	123 (67.2)
Age (years), mean (SD)	44.6 (8.6)
Age missing, *N* (%)	3 (0.0)
Number of years affiliated with the hospital, mean (SD)	13.2 (9.6)
Hospital years missing, *N* (%)	7 (0.0)
Number of years as quality manager, mean (SD)	4.6 (3.2)
Job years missing, *N* (%)	5 (0.0)

### Structure, reliability and validity

Table 2 gives an overview of factor loadings, Cronbach's alphas and corrected item-total correlations for each of the nine scales retained from factor analysis that were used to build the QMS index. These nine scales were quality policy documents (three items), quality monitoring by the board (five items), training of professionals (nine items), formal protocols for infection control (five items), formal protocols for medication and patient handling (four items), analyzing performance of care processes (eight items), analyzing performance of professionals (three items), analyzing feedback and patient experiences (three items) and evaluating results (six items). We eliminated 10 of the original 56 items in the questionnaire due to low factor loadings. As seen in Table 2, factor loadings ranged from 0.34 (‘benchmarking’) to 0.89 (‘professional training in quality improvement methods’), with most items achieving acceptable factor loadings (> 0.40). Confirmatory analysis supported this final structure (not reported here).

**Table 2:** Factor loadings, Cronbach's alpha and corrected item-total correlations (*N* = 181)

Scale and items	Factor loadings on primary scale	Internal consistency reliability: Cronbach's alpha	Corrected item-total correlation
*Quality policy documents*		0.75	
1. Written description of a formally agreed quality policy	0.817		0.655
2. Quality improvement plan at hospital level (translation of the quality objectives into concrete activities and measures designed to realize the quality policy)	0.847		0.718
3. Balanced score card (an overview of key quality measures focusing on clinical outcomes, finances, human resources and patient satisfaction)	0.424		0.381
*Quality monitoring by the board*		0.87	
1. The hospital (management) board makes it clear what is expected from care professionals in regards to quality improvement	0.780		0.730
2. The hospital (management) board has established formal roles for quality leadership (visible in organizational chart)	0.559		0.527
3. The hospital (management) board assesses on an annual or bi-annual basis whether care professionals comply with day-to-day patient safety procedures	0.791		0.739
4. The hospital (management) board knows and uses performance data for quality improvement	0.776		0.715
5. The hospital (management) board monitors the execution of quality improvement plans	0.870		0.809
*Training of professionals*		0.84	
1. Care professionals are trained by the organization to do their job	0.539		0.518
2. Care professionals are trained in teamwork	0.578		0.531
3. Middle management is trained in quality improvement methods	0.825		0.702
4. Care professionals are trained in quality improvement methods	0.889		0.781
5. Care professionals are trained in patient safety procedures	0.751		0.678
6. Care professionals follow at least one training session a year to further develop their professional expertise	0.509		0.479
7. Care professionals receive information back on the results of their treatment of patients	0.604		0.550
8. Care professionals are encouraged to report incidents and adverse events	0.507		0.456
9. Care professional licenses are reviewed by a regulatory body	0.316		0.304
*Formal protocols for infection control*		0.79	
1. Up-to-date hospital protocol for use of prophylactic antibiotics	0.489		0.435
2. Up-to-date hospital protocol for prevention of central line infection	0.698		0.626
3. Up-to-date hospital protocol for prevention of surgical site infection	0.789		0.705
4. Up-to-date hospital protocol for prevention of hospital-acquired infections	0.600		0.518
5. Up-to-date hospital protocol for prevention of ventilator-associated pneumonia	0.659		0.576
*Formal protocols for medication and patient handling*		0.77	
1. Up-to-date hospital protocol for medication reconciliation	0.643		0.564
2. Up-to-date hospital protocol for the handover of patient information to another care unit	0.661		0.576
3. Up-to-date hospital protocol for the use of medical aids (e.g. crutches, bandages, etc.)	0.683		0.597
4. Up-to-date hospital protocol for the prevention of medication errors	0.600		0.524
*Analyzing performance of care processes*		0.82	
1. Root-cause analysis of incidents (an incident is an unintended event that has cause or could cause harm to a patient)	0.687		0.623
2. Risk management (a systematic process of identifying, assessing and taking action to prevent or manage clinical events in the care process)	0.665		0.598
3. Internal audit (all components of the quality system are periodically assessed with regard to appropriate functioning, i.e. whether all procedures are adhered to and are effective)	0.543		0.476
4. Monitoring the opinions of care professionals (physicians and nurses are periodically asked about their satisfaction with their work, workload, the terms of employment, etc.)	0.545		0.497
5. Medical/clinical audit (various disciplines work together to assess and improve the results of care delivery)	0.573		0.517
6. Adverse event reporting and analysis (clinical staff is required to report and analyze all unexpected and preventable harm to patients caused by medical error or flaws in the healthcare system)	0.640		0.577
7. Systematic patient record review (systematic reviews of patient records are used to determine adverse events and priorities for quality improvement)	0.661		0.604
8. Development of care pathways/process redesign (all tests and treatments for a specific patient group are efficiently organized to delivery evidenced based care)	0.520		0.460
*Analyzing performance of professionals*		0.72	
1. Hospital (management) board ‘walk rounds’ to identify quality problems and issues (management visits work units to discuss quality and safety issues)	0.549		0.457
2. Monitoring individual physicians’ performance (physicians undergo systematic and documented performance assessments)	0.755		0.657
3. Monitoring individual nurses’ performance (nurses undergo systematic and documented performance assessments)	0.650		0.527
*Analyzing feedback of patient experiences*		0.48	
1. Benchmarking [specific results (indicators) are compared with other hospitals (best in class) in order to identify possible improvement]	0.336		0.219
2. Monitoring the options of patients (patients are periodically requested to give their opinions on the care provided; include surveys on patient views)	0.527		0.385
3. Complaints analysis (periodical evaluation of complaints is used to implement improvements)	0.452		0.289
*Evaluating results*		0.81	
1. Data used from clinical indicators to evaluate and adjust care processes	0.463		0.523
2. Data used from complication registration to evaluate and adjust care processes	0.568		0.629
3. Data used from incident reporting system to evaluate and adjust care processes	0.582		0.670
4. Data used from interviews/surveys with/among patients to evaluate and adjust care processes	0.578		0.634
5. Data used from assessment of guideline compliance to evaluate and adjust care processes	0.601		0.675
6. Data used from results of internal audits to evaluate and adjust care processes	0.626		0.726

Cronbach's alphas for internal consistency reliability were satisfactory for all scales (Cronbach's alpha = 0.72–0.87) except ‘analyzing feedback & patient experiences’ (Cronbach's alpha = 0.48). Based on the theoretical importance of feedback of patient experiences and benchmarking, we decided to keep this scale in the QMSI. The item-total scale correlations were acceptable within the range of 0.20 to 0.80. The correlation coefficients for items in the scale ‘feedback of patient experiences and benchmarking’ were consistently lower than those for the other scales. As shown in Table 3, the inter-scale correlation ranged from 0.11 (between ‘feedback of patient experience’ and ‘formal protocols for infection controls’) to 0.70 (between ‘evaluating results’ and ‘analyzing performance of care processes’). For all scales, each inter-scale correlation was below the pre-specified 0.70 threshold and deemed acceptable.

**Table 3:** Inter-scale correlation coefficients between the nine scales of QMSI (*N* = 181)

Scales	1	2	3	4	5	6	7	8	9
1. Quality policy documents	1								
2. Quality monitoring by the board	0.59	1							
3. Training of professional	0.40	0.66	1						
4. Formal protocols for infection control	0.27	0.20	0.14	1					
5. Formal protocols for medication and patient handling	0.37	0.41	0.43	0.57	1				
6. Analyzing performance of care processes	0.53	0.57	0.59	0.24	0.63	1			
7. Analyzing performance of professional	0.42	0.52	0.59	0.24	0.53	0.65	1		
8. Analyzing feedback of patient experiences	0.25	0.33	0.25	0.11	0.29	0.45	0.27	1	
9. Evaluating results	0.43	0.49	0.57	0.14	0.51	0.70	0.51	0.46	1

The numbers in the first row correspond with the scales in the first column.

The validity of the QMSI was further explored by analyzing its correlations with two other measures of quality management, namely the QMCI and the CQII. Correlation coefficients were within the acceptable range of 0.20 to 0.80 (Table 4).

**Table 4:** Correlations of QMSI with two other measures of quality in a subset of 74 hospitals that studied in depth

	QMCI	CQII
QMSI	0.48*	0.34*

*P < 0.05.

### Descriptive statistics for the QMSI and its scales

Descriptive statistics of items used to build the scales and the index are provided in Table 5. All items of the questionnaire were on a Likert-type response scale from 1 to 4. The average score on the individual items was ∼3, with a lower average score for items related to the analysis of the performance of professionals. Some floor and ceiling effects were found, where a high proportion of the respondents had a score at the lower or upper end of the answer categories, e.g. especially for patient complaint analysis and monitoring patient opinions. More than 80–90% of the hospitals had implemented the activities. The overall QMSI ranged from 0 to 27 points based on nine scales. The mean score of participating hospitals is 19.7 points (SD of 4.7).

**Table 5:** Descriptive statistics of the index and nine scales of the QMSI

Scale and items	Possible range	Average scores^a^	Floor (% with lowest score)	Ceiling (% with highest score)
*QMSI* (*N* = 181)^b^	0–27	19.7 (4.7)		
*Quality policy documents* (*N* = 179)	1–4	3.2 (0.8)		
1. Written description of a formally agreed quality policy	1–4	4 (1.0)	8	65
2. Quality improvement plan at hospital level (translation of the quality objectives into concrete activities and measures designed to realize the quality policy)	1–4	4 (2.0)	6	63
3. Balanced score card (an overview of key quality measures focusing on clinical outcomes, finances, human resources and patient satisfaction)	1–4	3 (2.0)	18	46
*Quality monitoring by the board* (*N* = 176)	1–4	3.2 (0.7)		
1. The hospital (management) board makes it clear what is expected from care professionals in regards to quality improvement	1–4	3 (1.0)	3	39
2. The hospital (management) board has established formal roles for quality leadership (visible in organizational chart)	1–4	4 (1.0)	8	66
3. The hospital (management) board assesses on an annual or bi-annual basis whether care professionals comply with day-to-day patient safety procedures	1–4	3 (2.0)	7	39
4. The hospital (management) board knows and uses performance data for quality improvement	1–4	3 (1.0)	4	44
5. The hospital (management) board monitors the execution of quality improvement plans	1–4	3 (1.0)	8	41
*Training of professionals* (*N* = 171)	1–4	3.2 (0.5)		
1. Care professionals are trained by the organization to do their job	1–4	4 (1.0)	2	59
2. Care professionals are trained in teamwork	1–4	3 (2.0)	4	26
3. Middle management is trained in quality improvement methods	1–4	3 (2.0)	4	30
4. Care professionals are trained in quality improvement methods	1–4	3 (2.0)	4	27
5. Care professionals are trained in patient safety procedures	1–4	3 (1.0)	1	40
6. Care professionals follow at least one training session a year to further develop their professional expertise	1–4	4 (1.0)	2	60
7. Care professionals receive information back on the results of their treatment of patients	1–4	3 (1.0)	2	47
8. Care professionals are encouraged to report incidents and adverse events	1–4	4 (1.0)	5	53
9. Care professional licenses are reviewed by a regulatory body	1–4	4 (1.0)	13	50
*Formal protocols for infection control* (*N* = 171)	1–4	3.5 (0.6)		
1. Up-to-date hospital protocol for use of prophylactic antibiotics	1–4	4 (1.0)	6	71
2. Up-to-date hospital protocol for prevention of central line infection	1–4	4 (0.0)	7	73
3. Up-to-date hospital protocol for prevention of surgical site infection	1–4	4 (0.0)	10	73
4. Up-to-date hospital protocol for prevention of hospital-acquired infections	1–4	4 (0.0)	3	82
5. Up-to-date hospital protocol for prevention of ventilator-associated pneumonia	1–4	4 (1.0)	11	54
*Formal protocols for medication and patient handling* (*N* = 170)	1–4	3.1 (0.8)		
1. Up-to-date hospital protocol for medication reconciliation	1–4	3 (2.0)	9	45
2. Up-to-date hospital protocol for the handover of patient information to another care unit	1–4	4 (1.0)	5	59
3. Up-to-date hospital protocol for the use of medical aids (e.g. crutches, bandages, etc.)	1–4	4 (2.0)	14	49
4. Up-to-date hospital protocol for the prevention of medication errors	1–4	3 (2.0)	13	48
*Analyzing performance of care processes* (*N* = 168)	1–4	2.9 (0.7)		
1. Root-cause analysis of incidents (an incident is an unintended event that has cause or could cause harm to a patient)	1–4	3 (2.0)	10	40
2. Risk management (a systematic process of identifying, assessing and taking action to prevent or manage clinical events in the care process)	1–4	3 (2.0)	12	35
3. Internal audit (all components of the quality system are periodically assessed with regard to appropriate functioning, i.e. whether all procedures are adhered to and are effective)	1–4	4 (2.0)	8	54
4. Monitoring the opinions of care professionals (physicians and nurses are periodically asked about their satisfaction with their work, workload, the terms of employment, etc.)	1–4	3 (2.0)	24	41
5. Medical/clinical audit (various disciplines work together to assess and improve the results of care delivery)	1–4	3 (2.0)	15	32
6. Adverse event reporting and analysis (clinical staff is required to report and analyze all unexpected and preventable harm to patients caused by medical error or flaws in the healthcare system)	1–4	4 (1.0)	5	60
7. Systematic patient record review (systematic reviews of patient records are used to determine adverse events and priorities for quality improvement)	1–4	3 (2.0)	8	45
8. Development of care pathways/process redesign (all tests and treatments for a specific patient group are efficiently organized to deliver evidenced based care)	1–4	3 (1.0)	15	22
*Analyzing performance of professionals* (*N* = 174)	1–4	2.6 (1.0)		
1. Hospital (management) board ‘walk rounds’ to identify quality problems and issues (management visits work units to discuss quality and safety issues)	1–4	2 (3.0)	27	34
2. Monitoring individual physicians’ performance (physicians undergo systematic and documented performance assessments)	1–4	2 (3.0)	37	34
4. Monitoring individual nurses’ performance (nurses undergo systematic and documented performance assessments)	1–4	3 (3.0)	26	41
*Analyzing feedback of patient experiences* (*N* = 174)	1–4	3.4 (0.5)		
1. Benchmarking [specific results (indicators) are compared with other hospitals (best in class) in order to identify possible improvement]	1–4	2 (2.0)	19	31
2. Monitoring the opinions of patients (patients are periodically requested to give their opinions on the care provided; include surveys on patient views)	1–4	4 (0.0)	2	82
3. Complaints analysis (periodical evaluation of complaints is used to implement improvements)	1–4	4 (0.0)	1	91
*Evaluating results* (*N* = 177)	1–4	3.1 (0.6)		
1. Data used from clinical indicators to evaluate and adjust care processes	1–4	4 (2.0)	3	52
2. Data used from complication registration to evaluate and adjust care processes	1–4	3 (2.0)	5	39
3. Data used from incident reporting system to evaluate and adjust care processes	1–4	4 (1.0)	6	61
4. Data used from interviews/surveys with/among patients to evaluate and adjust care processes	1–4	4 (1.0)	1	66
5. Data used from assessment of guideline compliance to evaluate and adjust care processes	1–4	3 (2.0)	14	28
6. Data used from results of internal audits to evaluate and adjust care processes	1–4	4 (1.0)	3	54

^a^Median (inter quartile range) presented for individual question items, mean (SD) presented for scales.

^b^QMSI is the sum of all nine scales (minus 9). This result includes data that have been subjected to multiple imputations.

## Discussion

We set out to develop and validate an index (QMSI) to measure QMSs in European hospitals. We found that the QMSI has 46 items to be reliable and is valid for the assessment of QMSs in European hospitals. The answers to the 46 items could be summarized in an index to express the extent of implementation of quality management activities, such as quality policies, methods for continuous improvement and procedures for patient complaint handling or staff education. The QMSI was found to be useful to differentiate between hospitals on nine separate scales and on the index as a whole. The nine scales of the QMSI represent the managerial aspects of quality management and leave room for the investigation of associations of quality management with leadership, patient and professional involvement and organizational culture. These latter concepts are assumed to influence the extent of implementation of quality management in hospitals.

### Comparison with earlier studies

The newly developed DUQuE instrument has good psychometric properties, consists of up-to-date questionnaire items, can be used in various European countries and is not too time-consuming for respondents (46 items; 9 dimensions). Earlier developed instruments have between 17 and 179 items, and 3–13 dimensions [1]. The DUQuE instrument for QMS covers four of the six domains found in the literature. Intentionally, the QMSI does not cover the domains leadership and patient involvement, because these are in the DUQuE framework influencing factors for the implementation of QMS and not part of the managerial aspects of quality management itself.

The clear sampling frame with random hospitals across EU countries has a higher external validity than existing research on QMS. In line with previous research, it seems that there is no individual focal area that accounts for the entire variance associated with the implementation of QMS. Quality management is a combination of policy, monitoring quality improvement by the board, professional development, monitoring of performance of processes and knowing relevant patient-related outcomes.

### Limitations of the study

This study has some limitations. The QMSI is based on the perception of the quality manager of the hospital. Although data from the questionnaire were self-reported, it has been shown through on-site visits that they seemed to be reasonably reliable. Despite the random selection of hospitals, selection bias among participating hospitals cannot be ruled out. Especially in some countries, the number of participating hospitals was smaller than that was initially planned for that country. Furthermore, the final study sample was too small to carry out a cross-culture validation. Therefore, further data collection and analysis will be needed before we can recommend the instrument for official use in cross-country comparisons. A positive point is that hospital and country coordinators did not report problems with the understandability or applicability of the questionnaire.

### Implications for research, policy and practice

As patients and purchasers expect the best possible quality of care, healthcare providers have to prove that they constantly work on quality improvement and safer healthcare. Our study has developed an efficient instrument to measure the implementation of quality management strategies on nine focal areas. We also kept some items with ceiling effects, which can still support policy-makers at EU-level stimulating the development of QMS in less-developed countries. Areas with floor effects, like monitoring physician performance, are recognized as important for the years to come and for further spread in European hospitals. The instrument and resulting index could be used for future comparative studies on quality management and for baseline assessment by hospitals or purchasers. A questionnaire is less time-consuming than a site visit and seems to give a quite reliable overall picture of the development and implementation of a QMS. Earlier research has shown that this kind of instrument is useful for monitoring implementation of QMSs over time [18]. More importantly, it can be used to test the assumption that enforcing certain quality management policies and strategies will lead to the desired effects, possibly linking management strategies to quality and safety outcomes. Forthcoming DUQuE work will lend additionally validity to the QMSI through the investigation of its relationships with other constructs and outcomes such as hospital external assessment, quality orientation of hospital boards, social capital, organizational culture, safety culture, clinical indicators and patient-reported experience measures.

## Conclusion

The newly developed and validated index (hence, instrument) of the implementation of QMSs presents an important tool for measuring, monitoring and, potentially, improving quality management in European hospitals. The QMSI is part of a broader group of instruments developed in the European research project ‘Deepening our Understanding of Quality improvement in Europe’ (DUQuE).

## Funding

The study, “Deepening our Understanding of Quality Improvement in Europe (DUQuE)” has received funding from the European Community's Seventh Framework Programme (FP7/2007-2013) under grant agreement n° 241822. Funding to pay the Open Access publication charges for this article was provided by European Community's Seventh Framework Programme (FP7/2007-2013) under grant agreement no. 241822.

## Appendix

The DUQuE Project Consortium comprises: Klazinga N, Kringos DS, Lombarts MJMH and Plochg T (Academic Medical Centre-AMC, University of Amsterdam, THE NETHERLANDS); Lopez MA, Secanell M, Sunol R and Vallejo P (Avedis Donabedian University Institute-Universitat Autónoma de Barcelona FAD. Red de investigación en servicios de salud en enfermedades crónicas REDISSEC, SPAIN); Bartels P and Kristensen S (Central Denmark Region & Center for Healthcare Improvements, Aalborg University, DENMARK); Michel P and Saillour-Glenisson F (Comité de la Coordination de l'Evaluation Clinique et de la Qualité en Aquitaine, FRANCE); Vlcek F (Czech Accreditation Committee, CZECH REPUBLIC); Car M, Jones S and Klaus E (Dr Foster Intelligence-DFI, UK); Bottaro S and Garel P (European Hospital and Healthcare Federation-HOPE, BELGIUM); Saluvan M (Hacettepe University, TURKEY); Bruneau C and Depaigne-Loth A (Haute Autorité de la Santé-HAS, FRANCE); Shaw C (University of New South Wales, AUSTRALIA); Hammer A, Ommen O and Pfaff H (Institute of Medical Sociology, Health Services Research and Rehabilitation Science, University of Cologne-IMVR, GERMANY); Groene O (London School of Hygiene and Tropical Medicine, UK); Botje D and Wagner C (The Netherlands Institute for Health Services Research-NIVEL, the NETHERLANDS); Kutaj-Wasikowska H and Kutryba B (Polish Society for Quality Promotion in Health Care-TPJ, POLAND); Escoval A and Lívio A (Portuguese Association for Hospital Development-APDH, PORTUGAL); Eiras M, Franca M and Leite I (Portuguese Society for Quality in Health Care-SPQS, PORTUGAL); Almeman F, Kus H and Ozturk K (Turkish Society for Quality Improvement in Healthcare-SKID, TURKEY); Mannion R (University of Birmingham, UK); Arah OA, Chow A, DerSarkissian M, Thompson CA and Wang A (University of California, Los Angeles-UCLA, USA); Thompson A (University of Edinburgh, UK).
